# Parents’ Awareness and Perception of Children’s Eye Diseases in Madinah, Saudi Arabia: A Cross-Sectional Study

**DOI:** 10.7759/cureus.22604

**Published:** 2022-02-25

**Authors:** Amal M Surrati, Sarah M Almuwarraee, Reem A Mohammad, Sarah A Almatrafi, Sara A Murshid, Lujain I Khayat, Hussain F Al-Habboubi

**Affiliations:** 1 Family Medicine, Taibah University, Medina, SAU; 2 Medicine and Surgery, Taibah University, Medina, SAU; 3 Ophthalmology, National Guard Hospital, Medina, SAU

**Keywords:** saudi arabia, children, eye diseases, eye care, amblyopia, awareness

## Abstract

Background

The visual system becomes more susceptible to conditions causing abnormal binocular interaction or blurred visual input as it matures during the first six years of life. Therefore, detection and treatment of visual impairment at a young age can lower the burden of the condition in adulthood. According to estimates, there are 19 million children worldwide with visual impairment, and 1.4 million children suffer from blindness. One of the strategies to address blinding eye conditions and visual impairment is through health promotion. For children, the efficient way of intervention is through parents and their school environment. Therefore, the establishment of an effective health promotion model for addressing childhood blindness requires awareness building of parents and health care practices. Few studies were undertaken in Saudi Arabia to focus on the awareness of parents about childhood eye diseases and visual impairment. The aim of this study is to assess parents’ awareness and perception of children’s eye diseases in Madinah, Saudi Arabia.

Methods

This cross-sectional study was conducted in Madinah, Saudi Arabia, from January to December 2021. A self-administered questionnaire was randomly distributed to parents of children aged 15 and younger via WhatsApp. The survey consisted of four main sections: socio-demographic data, knowledge about eye care, knowledge about eye diseases, and eye care practice.

Results

The knowledge about eye diseases in children was of excellent grade in 20 parents (3.6%), good in 101 (18.2%), and poor in 434 (78.2%). Knowledge was good in 287 parents (51.7%) for amblyopia, 65 (11.7%) for childhood cataract, and 69 (12.4%) for childhood glaucoma. The attitude regarding children wearing spectacles and undergoing ophthalmic surgery when needed was positive in 427 (76.9%) and 474 (85.4%) parents, respectively. Over half of the participants (58.6%) had visited an ophthalmology clinic for the examination of their children. Doctors, campaigns, and social media were the preferred modes of receiving knowledge. Participants aged 51 years or over (p = 0.022), with a higher income level (p = 0.004), of Saudi origin (p = 0.036), and those with a child with an eye disease (p = 0.001) had significantly higher knowledge scores about childhood eye diseases.

Conclusion

The levels of knowledge, practice, and attitude among parents regarding pediatric eye diseases were unsatisfactory. Health promotion through utilizing parents’ preferred modes of media could improve the eye care of children in the study area.

## Introduction

Visual impairment in children causes a sustained burden in adulthood and is, therefore, important to detect and manage at the early stages [1]. In the first six years of life, the visual system continues to mature, increasing its susceptibility to visual conditions that cause either blurred visual input or abnormal binocular interaction [2]. The worldwide prevalence of visual impairment in children is estimated to be 19 million, while the number suffering from blindness is estimated to be 1.4 million [3]. According to four studies performed in different cities in Saudi Arabia, it was found that refractive error, strabismus, and amblyopia were the most common causes of visual impairment among the pediatric age group, with minor differences in the order of prevalence [4-7].

The World Health Organization (WHO) and now the United Nations (UN) endorse the global effort to prevent blinding eye diseases and related visual impairment, and health promotion is one of the strategies to reach this goal [8]. Children are most easily reached through their parents and schools, since parents are usually the primary caregivers for them [9]. A health promotion model that successfully addresses childhood blindness must therefore include the awareness of parents and practices for managing eye diseases. Several studies have been conducted in India, Nigeria, and other countries [9-11], some of which have focused on specific eye diseases such as strabismus, myopia, or retinoblastoma [10,12,13]. In Saudi Arabia, multiple studies evaluating the awareness of adults regarding common eye diseases have been undertaken [14,15]. To the best of our knowledge, there have been very few studies conducted in Saudi Arabia that have focused on the awareness of parents about childhood eye diseases and visual impairment [16-18].

 The aim of this study is to assess the level of awareness and practice among parents and the determinants regarding eye diseases and visual impairments in their children in the Madinah region of Saudi Arabia.

## Materials and methods

This web-based survey was undertaken in January-December, 2021, after obtaining the approval of the ethical and research committee of Taibah University, Madinah, Saudi Arabia. The electronic informed consent of each participant was obtained. All tenants of the Helsinki declaration were strictly followed during all stages of research. Adult Saudi residents of the Madinah region with children aged under 15 were invited to participate in the survey. Those refusing to participate, living outside of Madinah, or working in a medical field related to eye care were excluded from the survey.

To achieve a 95% confidence interval with a 50% anticipated frequency of knowledge and 15% acceptable error margin, we needed at least 443 participants to be surveyed in the study area. We used OpenEpi software for calculating the sample size for the survey [19].

The Google platform for online surveys was used to collect participant information and responses. The questionnaire was divided into four parts. The first part covered the demographic data, including gender, age group, education, occupation, income, nationality, and if any of their children have an eye problem. The second part contained six questions regarding parents’ knowledge about eye care. The third part contained eleven questions on knowledge about childhood eye diseases. The fourth part contained six questions about eye care practice (Appendix 1).

The questionnaire was face validated by three consultants (two in ophthalmology and one in family medicine) and a pilot study was undertaken before distributing the questionnaire. Answers to each question were scored out of two points as follows: participants were given two points for each correct answer and zero points for incorrect answers. For questions with several correct answers, a participant was given two points if they chose all of the correct answers, one point if they chose at least one correct answer (even if other incorrect options were chosen), and zero points if they did not choose any correct answer. 

For each section of the questionnaire (knowledge about eye care, knowledge about eye diseases, and eye care practice), if the total score was 75% or more of the total possible score, the level of knowledge was considered to be excellent. Scores between 50% and 74% were considered good, and scores less than 50% were considered poor. The categories of good and excellent knowledge were combined to make the category acceptable.

The data was compiled using a Microsoft Excel® spreadsheet (Microsoft Corp., Redmond, Washington). It was then transferred to IBM SPSS Statistics for Windows, Version 19.0 (Released 2010. IBM Corp, Armonk, New York). Qualitative variables were presented as frequency and percentage, and quantitative data (scores) as the median and interquartile range. 

To compare the scores between the subgroups of participants, we used independent samples nonparametric tests (Mann-Whitney test for two independent samples and Kruskal-Wallis test for three or more independent samples). A two-sided p-value of <0.05 was considered to be statistically significant.

## Results

In total, 773 participants completed the survey. Of those, only 555 participants met the inclusion criteria and were eventually enrolled in the current study. More females (86.7%) participated than males (13.3%). One third (36%) of the participants were between 31 and 40 years, whereas only 7.7% of them were over 50 years. The surveyed population was mostly educated; 77.3% had a bachelor’s degree or above. Overall, 40.5% of parents reported an existing eye problem in their child. The demographic profile of participants is given in Table 1.

**Table 1:** Parents’ demographic data

Parameter	Subgroups	Number	Percentage
Age group (years)	20 to 30	135	24.3
	31 to 40	200	36.0
	41 to 50	177	31.9
	50 and over	43	7.7
Gender	Male	74	13.3
	Female	481	86.7
Education	School	82	14.7
	Diploma	44	7.9
	Bachelors	375	67.6
	Masters	54	9.7
Income (Saudi rial per month)	<5000	117	21.1
	5001 to 10,000	191	34.4
	10,001 to 20,000	211	38.0
	20,001 and over	36	6.5
Occupation	Employed	269	48.5
	Unemployed	249	44.9
	Retired	37	6.7
Nationality	Saudi	549	98.9
	Non-Saudi	6	1.1
Child with eye problem	No	330	59.5
	Yes	225	40.5

The parents’ responses regarding different eye diseases among their children are summarized in Table 2. One-third of the children of participating parents have a refractive error and one in twenty have amblyopia.

**Table 2:** Prevalence of different eye diseases among children as informed by parents in Madinah, Saudi Arabia.

Parameter	Frequency	Percentage
No eye problem reported	332	59.8
Refractive error (short-/long-sighted)	168	30.3
Amblyopia	32	5.8
Allergic conjunctivitis	7	1.3
Congenital cataract	3	0.5
Dryness	3	0.5
Nasolacrimal duct obstruction	2	0.4
Retinal disease	2	0.4
Strabismus	2	0.4
Facial nerve palsy	1	0.2
Congenital glaucoma	1	0.2
Chalazion	1	0.2
Color blindness	1	0.2
Total	555	100.0

The knowledge of parents about eye diseases in children was of excellent grade in 20 (3.6%), good in 101 (18.2%) and poor in 434 (78.2%) participants. The knowledge about amblyopia was acceptable in 325 parents (58.5%), childhood cataract in 84 parents (15.1%), and childhood glaucoma in 107 (19.2%).

The knowledge score is correlated with a number of determinants, shown in Table 3. Our data show that the knowledge score of eye diseases in childhood was significantly higher among participants aged 51 years and over (p = 0.022), with higher income level (p = 0.004), Saudi participants (p = 0.036), and parents who had a child with an eye disease (p = 0.001). On the other hand, gender (p = 0.264), educational level (p = 0.913), and occupation (p = 0.092) showed no significant effect on the knowledge score about eye diseases.

**Table 3:** Determinants of knowledge of eye diseases in childhood among parents of children in Madinah, Saudi Arabia IQR: interquartile range **A p-value less than 0.05 is considered statistically significant

Factors	Subgroups	Number	Median	IQR	p-value^**^
Gender	Male	74	6	7	0.264
	Female	481	7	6	
Age-group	20 to 30	135	7	5	0.022
	31 to 40	200	6.5	5	
	41 to 50	177	7	5.5	
	51 and over	43	8	5	
Education	School	82	7	6	0.913
	Diploma	44	7.5	5	
	Bachelors	375	7	6	
	Masters	54	7	7	
Income (SR per month)	<5000	117	6	8	0.004
	5001 to 10000	191	7	4	
	10,001 to 20,000	211	7	6	
	20,001 and over	36	9	7	
Occupation	Employed	269	7	6	0.092
	Unemployed	249	7	5	
	Retired	37	8	5	
Nationality	Saudi	549	7	6	0.036
	Non-Saudi	6	4	2	
Child with eye problem	No	330	7	5	0.001
	Yes	225	7	6	

**Figure 1:**
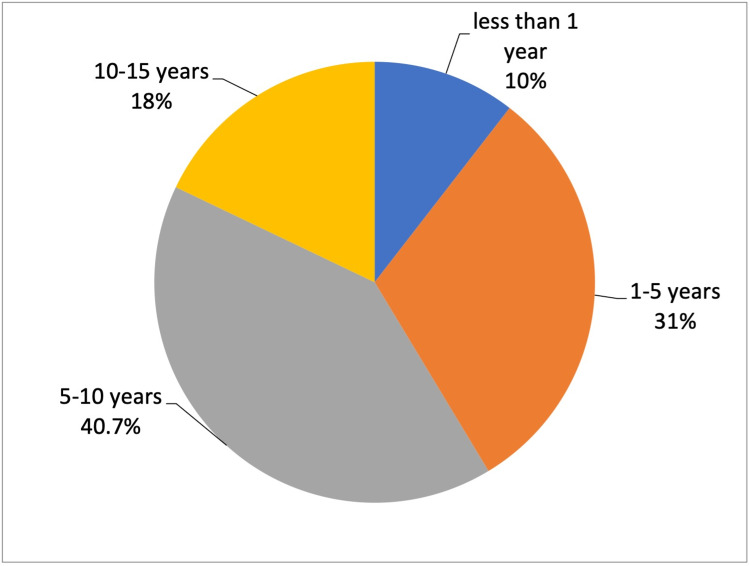
Age of the child when first taken for an eye exam

Over half of the participants (58.6%) reported that they had visited ophthalmology clinics for the examination of their children. The most common age at which a child was first taken to an eye exam is 5-10 years; other ages are summarized in Figure 1. 

In total, 41.4% of parents had never taken their child to an eye exam and the most common reason was due to the absence of signs that would prompt them to seek medical advice. Other reasons are summarized in Table 4.

**Table 4:** Parent’s reasons for not taking their child to an eye exam COVID-19: coronavirus disease 2019

Reasons	Frequency	Percentage
I didn’t notice any signs that would prompt me to take them to an eye doctor	169	30.5
I think my child is too young to have an eye test	33	5.9
I don’t know how and/or where to arrange an appointment for an eye test	26	4.7
I am worried that my child may be given glasses that they don’t need	16	2.9
I am worried about the cost of an eye test and glasses	8	1.4
No public awareness highlighting the importance of eye exams	2	0.4
Fear of my child being exposed to COVID-19	1	0.2

**Figure 2:**
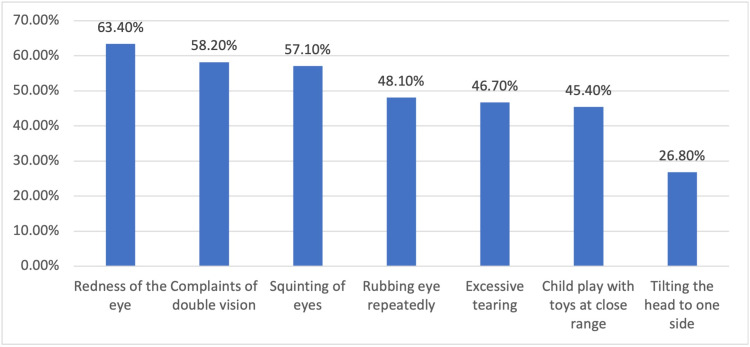
Observations that may prompt parents to take their child to an eye care practitioner

For further insight into the perceptions of parents toward pediatric eye exams, individuals were asked: ‘‘How frequently should a child receive an eye exam?’’ Of the total parents, 269 (48.5%) considered an annual eye exam beneficial for children, while 122 (22.2%) stated that an eye exam is beneficial ‘‘only when the child reports a problem”. Redness of the eye was the most distressing symptom that may prompt them to seek medical advice (Figure 2). 

The attitude of parents regarding wearing of spectacles by their child when needed was positive in 427 parents (76.9%), while that for ophthalmic surgery was positive in 474 (85.4%). Fear of the outcome (80.2%) was the most common reason for refusal of eye surgery (Table 5). 

**Table 5:** Parents’ reasons for refusal of ophthalmologic surgery in their children

Reason	Frequency	Percentage
Fear of outcome	65	80.2
It would cause more damage to the eyes	27	33.3
Cost of the actual operation	10	12.3
Accessibility of services	6	7.4
Cultural and social barriers	1	1.2

Doctors, internet browsers, social media, and friends were the parents’ current sources of information; however, the use of campaigns, television, and billboards were additional future modes they suggested.

## Discussion

The current study evaluated the level of knowledge, attitude, and practice of parents in Madinah, Saudi Arabia, regarding eye diseases in children. Parents with higher income, aged over 51, or who had a child with an eye disease had better knowledge of eye diseases in children than parents without these characteristics. The most common current source of information regarding eye diseases among parents were doctors, internet browsers, and social media.

The present study had a large sample compared to other studies in India and African countries [9,20]. Overall, most of the research done on this issue has shown reasonably good knowledge about different eye diseases in children, but their causes and management were poorly understood. Comparing outcomes of different studies is a challenge, as sample size, study design, and the prevalent causes of childhood blindness differed in different study sites.

In African countries and rural settings of developing countries, the lack of availability of services for child health care and health promotion results in less awareness among parents. The practice was also poor in terms of following the advice of optometrists/ophthalmologists regarding the use of optical aids and eye disease management [9,20]. 

In the present study, knowledge about childhood eye diseases was poor in 78.2% of parents. This is consistent with a previous study performed in the capital of Saudi Arabia with 1,070 participants, which showed a poor level of knowledge in 91.9% of parents regarding childhood eye diseases [17]. Similarly, Bashaar et al. noted an average knowledge score of 2 out of 7 among parents of children attending public schools [18]. Conversely, a study performed in Arar city showed that 56.7% of parents had a sufficient level of knowledge [16].

The poor level of knowledge in the current study may be attributed to the absence of campaigns related to eye health care. Ophthalmologists should be encouraged to undertake more campaigns and be more proactive in raising the level of knowledge among parents.

In the present study, the parents with a higher family income had better knowledge of eye diseases and care in children. Income may be an indicator of a higher level of education and access to health knowledge sources and health services in the private sector. Our data showed that higher education was significantly associated with higher monthly income (p=0.002) where 57.1%, 44.3%, and 30.5% of post-graduate degree holders, bachelor’s degree holders, and school graduates, respectively, reported receiving a monthly income of 10,000 Saudi Riyal (SAR) or more.

Life experience may also be an important predictor of knowledge, as older participants showed better knowledge in our study compared to younger parents. This is in accordance with data reported in 2019 from a study performed in Arar city [16].

There was significantly better knowledge of eye diseases and management among parents who had children with eye diseases compared to those without eye diseases. Frequent interactions with health professionals while seeking advice regarding the eye diseases of their children and trying to understand their child’s eye problem using the internet and social media may explain this improved level of knowledge. This difference has also been observed by other researchers [9,17]. In addition, parents may be concerned about having another child with an eye disease, meaning that they may be more knowledgeable about prevention and causes of childhood eye diseases.

The parents’ knowledge regarding amblyopia was the highest compared to other diseases. This is probably due to the fact that it was the most prevalent disease amongst children of participating parents. Amblyopia is one form of complication due to visual deprivation at early ages. Timely management of both amblyopia and its underlying cause is crucial. Parents often approach late, which may be due to lack of knowledge, lack of resources or negative attitude towards suggested modes of management. Knowledge of amblyopia in the present study was acceptable in 58.5% of participants, higher than the 30% of Saudi parents reported by Alsaqr et al [21]. In another study held in Jeddah, the western province of Saudi Arabia, the level of knowledge about amblyopia in children was sufficient in only 20% of participating parents [22]. In a country where preschool screening and school screening (as recommended by the WHO) is not yet well adopted, the high level of knowledge among parents regarding amblyopia and refractive errors is reassuring; however, public health organizations are urged to integrate primary eye care within the primary health care system in the study area to further improve knowledge. 

Based on our findings, knowledge regarding childhood cataract and glaucoma was of an acceptable level in 15.1% and 19.2% of participants, respectively. In the current study, only 26.8% of participants were aware that cataract can affect children. This is similar to a study performed in Jeddah, which showed a 35.7% level of awareness among parents [23]. To the best of our knowledge, no study has addressed the level of awareness about pediatric glaucoma in Saudi Arabia. Birth defects including congenital eye diseases are common among middle eastern populations [24,25]. Early detection and prompt management of cataract and glaucoma are therefore recommended to prevent visual disabilities among children. This will be possible if parents are aware about this condition and the detrimental consequences of late intervention.

Fortunately, poor knowledge among parents about eye diseases was not reflected in the level of practices for eye care of their children in the present study, with 58.6% of children having undergone eye examination. This is very similar to the results of another study, which noted that 52.6% of parents had taken their children for an eye examination [16]. The presence of both private and public eye facilities in the study area provides easier access to services and is reflected by the parents’ higher level of practice. 

Although the practice level is reassuring, it is not ideal, and strongly recommends the undertaking of a proactive vision screening initiative in the study area, including eye screening at birth, amblyopia screening and school screening. 

An additional important point to mention is that most parents who had never taken their child for an eye exam stated that the reason for not doing so was the absence of signs that would prompt them to visit an ophthalmologist. Redness of the eye, complaints of double vision, squinting, rubbing eyes repeatedly, excessive tearing, and playing with toys at a close range were the symptoms most parents stated would prompt them to visit an ophthalmologist. These symptoms are indeed cause to visit an ophthalmologist, but the misunderstanding that children should only visit after such problems arise, as opposed to periodically through checkups, can be corrected through health promotion. 

Another positive point regarding eye care practice is that 76.9% of parents accepted the wearing of spectacles by their children. This is in accordance with a previous study performed in Arar city, which showed an acceptance rate of 73% amongst parents [16]. However, Bashaar et al. noted an acceptance rate of only 42.3% amongst parents [18]. Regarding ophthalmologic surgery, 85.4% of parents in the present study accepted the idea, while another study showed an acceptance rate of 71.1% [18]. The positive attitude of parents regarding both usage of spectacles for their children and following ophthalmologists’ advice for eye surgeries reflects their cooperation in improving eye care for future generations and their trust in existing eye care in the region.

Health promotion is a key strategy to improve knowledge and eye care practices in the general population [8]. In the present study, the current sources of information regarding pediatric eye care amongst parents were mainly doctors, internet browsers and social media. In other studies, family and friends were the main sources of information [15-17]. It is well known that misinformation can be easily spread through social media, so it is important to correct these misunderstandings in future modes of health promotion by ensuring that information is only relayed by medical professionals. Perhaps one way to achieve this is through using parents’ preferred modes of information, such as campaigns and billboards, in addition to one-to-one counseling by doctors.

The implications of the present study are eye openers for public health, ophthalmologists and decision makers. There is a need to develop health education material targeting the parents of children without any eye diseases. There seems to be a difference between current and desired modalities of providing education. Highly prevalent conditions such as myopia, and related amblyopia and strabismus need to be proactively tackled through screening and integrating primary eye care for early detection and timely intervention as and when needed. Parents will be key in this initiative and their improvement in knowledge and attitude will help in improving their practice, in order to assist pediatric ophthalmologists in the management of these children. 

There were some limitations of this study. Since the survey was web based, all responses were from computer-literate parents. The awareness may therefore be overrepresented, as those who lack such skills are less likely to have exposure to information about childhood eye diseases. The study focused on childhood eye diseases of developed countries, so issues related to nutrition, lack of antenatal care and infections were not included in the questionnaire. Therefore, the prevalence of eye diseases found in present study should be compared to results from Latin American and African countries, and results interpreted with caution [26]. 

Determining the prevalence of childhood blindness is a challenge for logistical reasons and due to the low prevalence of eye diseases in children. However, indirect information about prevalent conditions from screening initiatives and review of the school health records could complement the findings of the present study. 

## Conclusions

The level of parents’ knowledge about common pediatric eye diseases as well as their practice regarding eye care in children in Madinah,Saudi Arabia is considered unsatisfactory. Children are greatly influenced by their parents as they are usually their first teachers, from whom they learn and adopt health habits. Therefore, parents’ awareness about eye diseases in children, their attitude towards seeking help, and management of diseases as per doctors’ recommendations are crucial in the preparation of health promotion materials. Newer modalities of communicating such health information that appeal to parents need to be adopted in the study area in order to improve their effectiveness.

## Appendices

Appendix 1: Research questionnaire 

Consent From Parents

Parents’ Awareness and Perception of Children’s Eye Diseases in Madinah, Saudi Arabia

We are a group of fifth-year medical students from Taibah University inviting you to participate in a scientific research that aims to assess parents’ awareness and perception regarding children's eye diseases in Madinah. 

This questionnaire will take approximately five to seven minutes to complete. Please note that all personal information will remain confidential and will only be used for the purpose of this study. Please also note that your participation is voluntary and you are able to refrain from participating or withdraw your data from the study at any time. For any inquiries you may have regarding the research, please contact us.

I agree that I have been informed of all aspects of the research and its objectives. 

I agree that my participation is voluntary and that I have been given the chance to inquire about the research. 

1.     Do you accept to participate in this study? 

-       Yes

-       No

2.     Do you have any children under fifteen years old?

-       Yes

-       No

3.     Do you live in Madinah? 

-       Yes 

-       If not, where do you live? ________

4.     Do you work in a field related to eye-health care? 

-       Yes 

-       No

Part 1: Parent Demographic Data

1.     Gender? 

-       Male

-       Female

2.     Age? 

-       20-30 years

-       31-40 years

-       41-50 years 

-       51-60 years

-       61-70 years

3.     Education level? 

-       Primary school 

-       Middle school 

-       High school

-       Diploma

-       Bachelor’s Degree

-       Master’s degree

4.     Occupation?

-       Employed

-       Unemployed

-       Retired 

-       Other ___________

5.     Income level? 

-       <5,000 SR

-       5000-10,000 SR

-       10,000- 20,000 SR

-       > 20,000 SR

6.     Nationality?

-       Saudi 

-       Other _________

Part 2: Parent Knowledge About Eye Care 

1.     Does your child have an existing eye problem? (If the answer is no, skip to question 5)

-       Yes 

-       No 

2.     If the previous answer was yes, which of the following eye diseases do they have?

-       Refractive error (short sight/ farsighted) 

-       Amblyopia 

-       Congenital glaucoma 

-       Congenital cataract

-       Other ………………..

3.     In regard to the visually impaired child, do they attend school?

-       Yes 

-       No 

4.     If the previous answer was no, why? 

-        Don’t know where to send child and/or children for schooling

-        Without seeing, you cannot be educated

-        Scared that the child and /or children will not cope

-        Child and/ or children will be teased by peers

-        Other ………………….

5.     Please read and respond (select) with your existing knowledge to the following statements:

-    Wearing glasses if you need them when under seven years of age will make your eyes and vision develop normally 

a)    Agree

b)    Disagree   

c)    Not Sure

-    It is normal for a child aged one to seven years to occasionally have an eye turn 

d)    Agree

e)    Disagree   

f)     Not Sure

6.      What is your source of information regarding eye disease? (You can select more than one choice)

-       Nurse

-       Doctor

-       Neighbor

-       Friends

-       Social media

-       Television

-       Internet browsers

-       Radio

-       Others

7.     Preferred option to spread information about children’s visual problems? (You can select more than one choice) 

-       Nurse

-       Doctor

-       Campaigns 

-       Billboards 

-       Traditional healer

-       Social media

-       Television

-       Internet browsers

-       Radio

-       Others

Part 3: Knowledge About Common Eye Diseases

1.      What is the definition of amblyopia?

-       Decreased vision in one or both eye

-       Degeneration of optic nerve

-       Decrease night vision

-       Misalignment of both eyes

-       I don’t know

2.      What are the causes of amblyopia?

-       Refractive error

-       Strabismus

-       Cataract

-       TV and smart devices

-       Hereditary

-       Fever in infancy 

-       I don’t know

3.      What is the treatment of amblyopia?

-       Patching the strong eye 

-       Glasses 

-       Eye muscle exercise

-       Surgery and laser

-       No need for treatment

-       I don’t know

4.      Regarding treatment outcomes of amblyopia, does early treatment lead to better outcomes? 

-       Yes

-       No

-       I don’t know

5.      Which of the following is considered a complication of amblyopia?

-       Decreased visual acuity

-       Blindness

-       Disability

-       Impaired quality of life

-       School failure

-       I don’t know

6.      What is a cataract?

-       A white spot in the eye.

-       A lens changes where the lens becomes opaque.

-       A white membrane growing over the eye.

-       I don’t know

7.      Does cataract affect children?

-       Yes

-       No

-       I don’t know

8.      Can cataract lead to permanent blindness in children?

-       Yes

-       No

-       I don’t know

9.      What is glaucoma?

-       High pressure in the eye.

-       A damage to the nerve of the eye due to high pressure.

-       An age-related process leading to decrease in peripheral vision.

-       I don’t know

10.   Can glaucoma affect children?

-       Yes

-       No

-       I don’t know

11.   Can congenital glaucoma lead to blindness?

-       Yes

-       No

-       I don’t know

Part 4: Practices About Eye Care

1.     Has your child ever had any kind of eye or visual test?

-       Yes 

-       No 

2.     If the previous answer was yes, what was the age of the child when he/she was first taken to an eye exam?

-       Less than one year

-       1-5 years

-       5-10 years

-       10-15 years

3.     If the previous answer was no, what were the reasons? (you can select more than one choice) 

-       I don’t know how and/or where to arrange an appointment for an eye test

-       I am worried about the cost of an eye test and glasses 

-       I think my child is too young to have an eye test

-       I am worried my child may be given glasses he/she doesn’t need 

-       I didn’t notice any signs that would prompt me to take him to an eye doctor.

4.     How frequent should a child have a routine eye exam? 

-       Every year 

-       Every two years 

-       Every five years

-       Only when the child reports a problem 

-       Not sure

5.     What kind of observation might prompt you to take your child to an eye care practitioner? (You can select more than one choice) 

-       Child playing with toys at close range

-       Rubbing eye repeatedly

-       Tilting the head to one side

-       Squinting of eyes

-       Complaints of double vision

-       Excessive tearing

-       Redness of the eye

6.     Do any of your children wear glasses? 

-       Yes 

-       No 

7.     If the previous answer was yes, do you know what they are for?

-       Refractive Error

-       Amblyopia 

-       Cataract

-       Strabismus

-       I'm not sure what they are for 

-       Other ___________

8.     Do you accept the idea of your child wearing spectacles?

-       Yes

-       No

-       I'm not sure

9.     Would you allow your child to undergo eye surgery if needed? 

-        Yes

-        No

10.  If the previous answer was no, why? 

-        Fear of outcome

-        It would cause more damage to the eyes

-        Cost of the actual operation

-        Cultural and social barriers

-        Accessibility to services
